# Cardiac rehabilitation patients experiences and understanding of group metacognitive therapy: a qualitative study

**DOI:** 10.1136/openhrt-2021-001708

**Published:** 2021-07-14

**Authors:** Rebecca McPhillips, Lora Capobianco, Bethany Grace Cooper, Zara Husain, Adrian Wells

**Affiliations:** 1 Social Care and Society, Division of Population Health, Health Services Research and Primary Care, School of Health Sciences, Faculty of Biology, Medicine and Health, The University of Manchester, Manchester, UK; 2 Research and Innovation, Greater Manchester Mental Health NHS Foundation Trust, Manchester, UK; 3 School of Psychological Sciences, Faculty of Biology, Medicine and Health, Manchester Academic Health Science Centre, The University of Manchester, Manchester, UK

**Keywords:** anxiety, depression, cardiac rehabilitation

## Abstract

**Objective:**

Depression and anxiety are up to three times more prevalent in cardiac patients than the general population and are linked to increased risks of future cardiac events and mortality. Psychological interventions for cardiac patients vary in content and are often associated with weak outcomes. A recent treatment, metacognitive therapy (MCT) has been shown to be highly effective at treating psychological distress in mental health settings. This is the first study to explore qualitatively, cardiac rehabilitation (CR) patients’ experiences and understanding of group MCT with the aim of examining aspects of treatment that patients experienced as helpful.

**Methods:**

In-depth qualitative interviews were conducted with 24 purposively sampled CR patients following group MCT. Data were analysed using thematic analysis.

**Results:**

Two main themes were identified: (1) general therapy factors that were seen largely as beneficial, where patients highlighted interaction with other CR patients and CR staff delivery of treatment and their knowledge of cardiology; (2) group MCT-specific factors that were seen as beneficial encompassed patients’ understanding of the intervention and use of particular group MCT techniques. Most patients viewed MCT in a manner consistent with the metacognitive model. All the patients who completed group MCT were positive about it and described self-perceived changes in their thinking and well-being. A minority of patients gave specific reasons for not finding the treatment helpful.

**Conclusion:**

CR patients with anxiety and depression symptoms valued specific group MCT techniques, the opportunity to learn about other patients, and the knowledge of CR staff. The data supports the transferability of treatment to a CR context and advantages that this might bring.

**Key questions**

**What is already known about this subject?**

- Anxiety and depression are up to three times more prevalent in people with cardiovascular disease (CVD) than in the general population and are linked to negative outcomes for patients with CVD including poorer treatment adherence and quality of life, increased use of healthcare, risk of future cardiac events and mortality.
- Cardiac rehabilitation (CR) is widely considered a vehicle for psychological support, with techniques including relaxation training, stress management and elements of cognitive–behavioural therapy all being used to promote the mental health of patients with CVD. However, patients are often reluctant to talk about their concerns in the context of CR and the above techniques can be viewed as superficial. More effective psychological interventions that can be integrated into CR and are acceptable to patients are required.

**What does this study add?**

- This study investigated the subjective value of a recently developed treatment, group metacognitive therapy (group MCT), for CR patients suffering with anxiety and depression. The majority of patients viewed the group modality and CR staff delivery of the intervention as positive and had an understanding of group MCT that was consistent with the metacognitive model. A small proportion of patients inaccurately interpreted group MCT as being concerned with problem solving and positive thinking. Patients who completed the intervention recounted using group MCT techniques and discussed self-perceived positive changes to their emotional well-being.
- Only 4 out of 24 patients did not complete the intervention. Two of these patients provided specific reasons as to why they did not find group MCT helpful. They explained that they did not feel the intervention addressed their needs and they were critical of CR staff delivery.

**Key questions**

**How might this impact on clinical practice?**

- In the context of increasing calls for the integration of physical and mental health services this study indicates that group MCT is suitable for cardiac patients and supports the transferability of the intervention to physical health contexts and delivery by non-specialists. Patient accounts point to areas to monitor to avoid drift in understanding, notably around misinterpretations of the aims of MCT and the incorrect use of the techniques involved.

## INTRODUCTION

Cardiovascular disease (CVD) presents a huge burden of disease globally.^1 2^ However, in the past three decades, there has been a significant decline in deaths caused by CVD in all high-income and some middle-income countries.^1^ Consequently, there has been an increase in the number of people living with CVD. Depression and anxiety are among the most common comorbidities in CVD and are up to three times more prevalent in people with CVD than in the general population.^3 4^ In the UK, 28% of cardiac patients report borderline or clinically significant levels of anxiety when starting cardiac rehabilitation (CR), and 19% report borderline or clinically significant levels of depression.^5^ Depression and anxiety in those with CVD are linked to poorer treatment adherence and quality of life, increased use of healthcare, risk of future cardiac events and mortality.^6–8^ CR is widely considered a means for psychological support using techniques such as relaxation training, stress management and cognitive–behavioural therapy (CBT).^9–12^ Qualitative studies have investigated types of psychological support cardiac patients would prefer, which include talking therapies and group-based interventions, rather than antidepressants.^13 14^ In practice, however, CR staff and patients report minimal discussion of emotional needs, with patients being reluctant to talk about their worries and dismissive of guided relaxation and stress management techniques as these are viewed as superficial.^14 15^ Meta-analyses of current psychological interventions for cardiac patients show high variability, low study quality and often weak outcomes.^16 17^ It is evident that more effective psychological interventions and higher-quality trials are needed.

Advances in mental health treatment might offer translational opportunities for treating psychological distress in CR patients. A recently developed treatment, metacognitive therapy (MCT)^18 19^ is highly effective in mental health settings. A meta-analysis evaluating the efficacy of MCT for anxiety and depression found that MCT produced large post-treatment effect sizes compared with waitlist control (hedges g=2.06).^20^ When MCT was compared with cognitive and behavioural interventions there were large effect sizes favouring MCT at post treatment (hedges’ g=0.69). MCT is based on an information processing model^21 22^ of psychological disorder in which distress is maintained by a maladaptive thinking style called the cognitive attentional syndrome (CAS). The CAS is characterised by difficult to control repetitive negative thinking (ie, worrying, rumination, dwelling on events), inflexible attention and maladaptive coping strategies (eg, avoidance, thought suppression) and is linked to underlying positive and negative metacognitive beliefs. Positive metacognitive beliefs concern the usefulness of worrying, for example ‘worrying means I’m prepared’, while negative metacognitive beliefs concern the harmfulness and uncontrollability of overthinking, for example ‘worrying will cause me to have a heart attack’, ‘I can’t stop worrying’. Pilot studies suggest that MCT is a feasible treatment that can be effective in physical health and in anxiety and depression in CR patients in particular.^23^

While quantitative research can establish the efficacy of psychological interventions the focus is limited to establishing whether there is a causal relationship between an intervention and patient change. Quantitative methods cannot provide insights into how complex interventions such as MCT are experienced or understood by patients.^24 25^ Qualitative methods are designed to facilitate insight into patients’ experiences and understanding of complex interventions and can provide information on how treatments are received, addressing questions concerning the appropriateness of the intervention, why it was successful or not, and any variation in effectiveness in the sample.^24^ Thus, qualitative methods are advocated for the understanding and transferability of complex interventions.^26 27^

This qualitative study was conducted as part of the National Institute for Health Research funded PATHWAY trial, which compared group MCT plus CR to CR alone for psychologically distressed CR patients.^23 28^ We interviewed patients in the intervention arm when they had completed group MCT. Our aims were to explore patients’ experiences and understanding of group MCT. In particular, we wanted to understand whether and how patients had engaged with and used the different techniques and what they valued in the treatment.

## Methods

### Participants

Patients referred to three CR services in England were screened by CR staff for eligibility for the PATHWAY trial. Details on the inclusion and exclusion criteria can be found elsewhere.^28^

Patients who were recruited to the trial were asked at the point of entry if they consented to be contacted about qualitative research. Out of the first 79 patients who enrolled in the trial, 77 agreed to be contacted. Sixty patients from both the control and intervention arms of the trial were purposively sampled to include women and men with differing levels of distress and were approached by telephone by RM. Of which 43 patients from the control and intervention arms of the trial provided written informed consent and took part in time point 1 (T1) interviews, during CR but before group MCT, the results of which are published elsewhere.^15^ For the current study only patients allocated to the intervention arm were eligible (n=32). Patients who attended ≥4 group MCT sessions were defined a priori as completing the intervention. Of the 32 intervention patients who took part in T1 interviews, 22 completed the intervention, and 20 took part in time point 2 (T2) interviews and were included in the analysis. Of the 10 who did not complete the intervention, five completed T2 interviews. Of these five patients, one did not attend any group MCT sessions and subsequently this transcript was not included in our analysis. The remaining four transcripts were retained as these patients attended ≤2 group MCT sessions, and these transcripts provide important information in relation to the aims of this study (see online supplemental table 1). Thus, a total of 24 patients were included in the analysis (20 Group MCT completers, 4 non-completers).

10.1136/openhrt-2021-001708.supp1 Supplementary data

### Group MCT

Patients in the intervention arm of the trial were invited to take part in six group MCT sessions, each lasting 60–90 min, held at their CR centre. Sessions were delivered by CR staff, who were trained and supervised in the delivery of manualised group MCT. Sessions focused on developing a case formulation, socialisation to the model, modifying negative metacognitive beliefs and developing a helpful behaviours prescription. Participants were introduced to specific techniques such as the Spatial Attentional Control Exercise (SpACE), detached mindfulness, and worry and rumination postponement, which aimed to help patients develop control over perseverative thinking and to challenge metacognitive beliefs. Patients’ metacognitive beliefs were measured using ‘belief thermometers’ on a scale of 0–100 at each session, 100 indicating strong agreement with a metacognitive belief and 0 indicting no agreement with a metacognitive belief.

### Procedure

The patients in this study were all interviewed at T1 and T2 by RM, in their homes, at RM’s office, at their CR centre or in a public café as per patient request. Interviews were conversational in nature. Topic guides containing open questions and prompts were used to encourage patients to share their experiences in their own words, and closed questions were used to probe specific points. During T1 interviews patients were asked about their emotional experiences since their cardiac event.^15^ Data gathered at T1 was used to inform T2 interviews, during which patients were asked about their emotional experiences since T1, their views and experiences of group MCT, and whether and how they had engaged with and used the techniques they had learnt. RM tailored questions for individuals by asking, for example, if they still worried about situations they described worrying about at T1 and drew on the specific concerns that patients had discussed during T1 interviews when asking patients to explain how they had used, or would use, group MCT techniques. Interview guides were modified iteratively, as interviews and data analysis proceeded, to test developing ideas. Interviews ceased when the researchers agreed that interviews conducted later during the study were not generating any different information to those conducted earlier on. Interviews lasted between 14 and 88 min (mean 52 min).

Interviews were audiorecorded, transcribed verbatim and pseudonymised. RM led the thematic analysis.^29^ RM refamiliarised herself with the data by rereading each transcript, following which she coded data within each transcript that was relevant to the aims of the study. RM, LC and AW then discussed the codes that had been generated and discrepancies were resolved during discussion. RM, BGC and ZH reviewed the coded data and collated extracts into candidate themes, ensuring that themes met Patton’s internal homogeneity and external heterogeneity criteria.^30^ Candidate themes were discussed by all authors, and on agreement semantic themes were identified.

## Results

Two main themes were identified in relation to how patients discussed their experiences and understanding of group MCT. The first main theme concerned general therapy factors that were central to patients’ experiences of treatment, and the subthemes within this main theme concerned interaction with other CR patients and CR staff delivery of the intervention. The second main theme concerned MCT-specific factors that were dominant in patients’ experiences and understanding. The subthemes identified within this main theme were patients’ perceptions of the aims of group MCT, experiences of individual techniques taught during group MCT and perceptions of the effectiveness of group MCT. Within each of these subthemes, there were patients whose accounts were closer to the metacognitive model than others and these differences are highlighted within the analysis.

### Main theme 1: general group MCT factors

The first main theme encompasses two general therapy factors, interaction with other CR patients and CR staff delivery of group MCT that were dominant in patients’ accounts of their experiences of group MCT. These factors were seen as largely beneficial and the majority of patients reflected that the group format and CR staff delivery facilitated their engagement with the intervention.

#### Interaction with other CR patients

Interaction with other CR patients was central to patients’ accounts of their experiences of group MCT. Most patients found reassurance in talking to other CR patients as this allowed them to normalise their feelings, as P07 explained, ‘[Group-MCT] made me feel that it’s not unusual… you’re normal… everyone goes through a similar range of experiences’.

Group MCT provided a positive environment where patients felt comfortable to talk about their thoughts, which they may otherwise have kept to themselves:

‘It was good to get it out…you opened up a bit once you started talking… I found that really helpful… they brought things out you’d keep deep down in you’[P09]

Patients also explained that they believed that interacting with other patients went beyond reassurance and facilitated their taking part in the intervention itself, as one patient explained that working with other patients when taking part in a group exercise designed to challenge metacognitive beliefs was useful as, ‘we fired off each other’[P23], and another patient talked about the reduction in her groups’ scores on belief thermometers:

‘We started off with very high scores and they came down [Interviewer: was it useful to see the scores coming down?] Yes’[P16].

Only three patients talked about interacting with other CR patients as part of group MCT negatively. Two patients felt that there was too much time wasted when patients were discussing their concerns, ‘before you knew it, you’d spent ten, fifteen minutes, listening to one person telling the reasons why they feel like they do’ [P17]. The other explained that they did not want to talk about their concerns with a group, ‘I didn’t want to seem weak… everyone else was exposing their weaknesses’[P15].

Interestingly, three patients commented on small group size as being a factor that negatively impacted their experience of group MCT, ‘we’re suffering because there’s only two of us’[P05], ‘you probably do need about three or four [patients] just to spring off each other’[P03]. This highlighted the importance of a minimum of three to four patients to optimise patient experience.

#### CR Staff delivery of group MCT

The other general factor that was dominant in patients’ accounts of group MCT was its delivery by CR staff. Predominantly, patients described the delivery of group MCT positively, referring to staff as ‘very patient’[P01], and ‘embracing and friendly’[P15]. Patients explained that CR staff had enabled them to feel relaxed enough to talk, even when they had reservations about doing so at the start:

‘I was hung up about standing up like at AA… it wasn’t like that… it was alright because the cardiac rehab nurses I knew anyway, it wasn’t like talking to a stranger… so it wasn’t a big issue talking in the group… they care about you out of therapy anyway… you could speak to them about anything’[P10].

Patients also found reassurance in talking to CR staff, ‘you talk to the nurses, they say ‘that’s perfectly normal, don’t worry about that’… they’re there just to reassure you’[P07].

For some patients, the specific expertise of CR staff was understood to be of central importance in their ability to deliver group MCT in the context of CR, ‘the people [CR staff] presenting it [Group-MCT]… because they have looked after us through our body we have faith in their ability’[P05], ‘it’s important not just that they know about how to give this therapy, but also that they’ve got an awareness of people’s hearts’[P03].

Patients commented on staff referring to the group MCT manual and asking each other questions, and for most this was described as aiding engagement with group MCT. One patient commented about staff using the manual and said ‘it’s nice to know that other people are fallible’[P15], while another patient stated

‘I think because they ask each other questions, they give you permission to open up completely and be as honest as you could be, which is a good thing’[P14].

In contrast, the two patients who had attended only 2 sessions of group MCT, were negative about CR staff delivery of the intervention and criticised a perceived lack of knowledge and style of delivery:

‘On the first session she [CR staff] was actually one finger on the manual, reading it out… it was as if she’d got no knowledge of what she was doing[P24].

### Main theme 2: group MCT-specific treatment factors

The second main theme concerns three group MCT specific factors, patient perceptions of the aims of group MCT, patients’ experiences of using group MCT techniques and the changes they perceived in themselves as a result of treatment. The majority of patients had an understanding of the aims of MCT that was consistent with the metacognitive model and were positive about the techniques used. All patients who completed the intervention described the experience positively and reflected on self-perceived changes to their thinking and well-being.

#### Patient perceptions of the aims of group MCT

All the patients were asked what they thought group MCT was about. Most patients explained that group MCT was about ‘understanding the way you are thinking’[P20], ‘just acknowledging that you have this thought, but you’re not gonna analyse it too deeply’[P07]. These patients explained that as a result of group MCT they could see how perseverative or repetitive thinking was the cause of their distress:

‘Worry’s just a thought and it’s only in your mind, but it makes you feel ill… so obviously if you can control the worrying you’re not gonna feel as down or ill’.[P19]

For other patients accounts of the aims of group MCT were not as closely aligned with the metacognitive model. In these cases patients, most often explained that group MCT was about positive thinking, ‘putting… negativity into positivity’ [P16], and problem solving, ‘just trying to work out what was causing the worry’ [P13] although group MCT does not use either positive thinking or problem solving to address emotional distress. One patient’s explanation of the aims of group MCT combined positive thinking with being able to control thinking:

‘[Group-MCT] makes you look at your way of living and your beliefs and questioning whether they are helpful or not… it’s about whether you can control your thoughts, and if you can control them, how to make them positive and not negative’.[P15]

Patients who had experience of other psychological interventions drew on the contrasts between them and group MCT. One contrasted group MCT and CBT:

‘Not like with CBT, you are not told to change your thoughts are you in this therapy… and also you’re not told that it’s wrong to feel the way that you have been feeling, it’s suggesting you have got another option… it’s okay to feel that way but you don't have to let it drag you down and, you know, make you anxious’.[P04]

Another patient contrasted group MCT to counselling:

‘Because they’re not asking you to relive your past or bring things up that you don’t want to talk about… [Group-MCT] teaches you how to not have to worry, or worry as much, or feel anxious and scared, so that was good, it wasn’t dragging stuff up’.[P17]

Two of the patients who did not complete group MCT were negative about what they understood the aims of the intervention to be. One patient explained his understanding of group MCT to be ‘concentrated on unwanted thoughts, as though it was trying to find out something like… are you thinking suicidal’ [P12] and did not feel that the intervention was suitable for him as a result. The other patient explained that he believed the usefulness of group MCT ‘depend(ed) on how big a worry is’, explaining that ‘I wasn’t able to switch off about my [cardiac test] results’ and ‘you’ve got to worry about how you’re paying for things and not having money’ [P24]. Both patients expressed frustration with group MCT because they did not feel it was tailored to their needs overall, and P12 did not continue to attend because he ‘expected them [sessions] to be a little bit more intense than they were… there didn’t seem to be a lot of progress in each session’.

#### Patient experiences of using group MCT techniques

The techniques SpACE, detached mindfulness and worry postponement were central to patients’ accounts of their understanding and experience of treatment. Patients who had come to realise that repetitive thinking caused their distress described using the techniques as intended, to disengage perseverative thinking, ‘[SpACE] helps you [realise] … because something comes in your mind doesn’t mean you have to think about it’[P22]. Another patient reflected on how detached mindfulness helped him to relate to thoughts, ‘you might recognise a trigger… something that previously [would have started] a train of thought, you think, ‘well okay, I’m just gonna accept that, I’m not gonna dwell on it’’, and he drew on this when practicing worry and rumination postponement:

‘Postponement, that’s a good thing… you think, ‘oh I can’t deal with that, I’m gonna deal with that next Thursday’… for me that works… you sort of hung that problem on a hook and you move on… and it doesn’t come back into your head I found’.[P07]

These patients also explained that using group MCT techniques required practice to understand the benefits, ‘when you first start the SPACE… I thought you try to push thoughts away, whereas further down the line, you start to let them in and out… letting them go’.[P09]

Patients whose accounts of group MCT were around positive thinking and problem-solving described ways in which they used the techniques somewhat differently to what was intended. In this group, SpACE was often described as being used as a relaxation exercise, ‘if I do get very stressed, I go on the tape [SpACE]… and it makes me very very relaxed’[P17]. These patients (inappropriately) applied techniques such as SpACE to control perseverative thinking and maintained a sense of no control, as one patient explained:

‘The thought comes into your head and then you let that worry you… they’re teaching you to control that… which I thought was a good thing, but I said to them… when I’ve sorted the house, when we’ve moved… once I’ve sorted my problems out I can see how that [Group-MCT] is gonna be miles more beneficial’.[P21]

These patients often talked about detached mindfulness and worry postponement as techniques they could use to push thoughts away, which in the metacognitive model is classified as a maladaptive coping strategy.^18^ One patient explained:

‘[Group-MCT is about] going over worrying thoughts and how to postpone those thoughts… how to push ‘em away and try to get to a certain percentage of positive thinking’.[P06]

#### Self-perceived changes as a result of group MCT

All the patients who completed the intervention described self-perceived changes and improvements in their thinking, emotions and well-being. Patients explained that, ‘[Group-MCT] has actually changed the way I think, the worries… it’s really taught me you can’t sit there… thinking you are going to have another one [myocardial infarction] … you’ve gotta carry on, get out there’ [P09]. Patients not only valued the self-perceived changes they had already experienced as a result of group MCT, but also as something to draw on in future:

‘I just feel dead privileged that I can just like draw on it whenever I want… So it’s there and as long as I need it and I want to use it, which I will do… so it is not just six weeks, it’s kind of like life-changing for me’. [P04]

## Discussion

This study aimed to explore CR patients’ experiences and understanding of group MCT to discover what was found to be helpful or unhelpful and the accuracy of the message extracted. The general factors that were identified highlighted the benefits of interaction with other CR patients and CR staff delivery. For the vast majority of patients, the group format was described positively and can be understood to facilitate the treatment. In addition, CR staff delivery of the intervention was a positive factor for many patients, as staff were described as providing an environment which felt reassuring, and patients felt confident to take part. A minority of patients were disparaging about CR staff delivery of the intervention, due to their reliance on the manual. While some patients appreciated that staff were adhering to a manual, others felt as though it detracted from the experience. As delivery of group MCT was being evaluated as part of a randomised clinical trial adherence to the study manual and protocol was important, even though CR staff’s adherence to a protocol appears to have detracted from some patients’ experience. Despite this, patients appeared to value the knowledge of cardiology that CR staff brought to sessions. These results are mainly positive and indicate that cardiac staff, who are non-specialists in mental health, established and maintained a therapeutic alliance that was valued by patients.

Accounts of the aims of group MCT varied, from those that were consistent with the metacognitive model to those that were ‘off model’. However, where patients correctly understood that the aim of therapy was to disengage repetitive thinking their accounts appeared to show they had developed a greater sense of flexibility in their reaction to worry.

Many patients described using the techniques taught as intended, to flexibly disengage from repetitive thinking. However, a small number of patients described using these techniques in a way that was not consistent with the treatment model, as relaxation exercises and techniques that could be used to push thoughts away. All the patients who completed the intervention were positive about these techniques and described self-perceived changes and improvements in their thinking, emotions and well-being as a result of the intervention. The results suggest that the techniques of MCT are largely understood and used as intended and that they are experienced as positive. However, therapists should remain alert to potential misinterpretation and inappropriate use of the techniques as relaxation and thought suppression/avoidance techniques. Thus, group MCT might be augmented by further exercises that draw contrasts between SpACE and relaxation and between detached mindfulness and thought control/distraction.

Some patients also contrasted their experience in group MCT with other therapies, highlighting that unlike CBT, group MCT did not require patients to change their thoughts. Despite the fact that MCT does not aim to challenge or deal with worry content, patients did not generally see this as a barrier to participation. Only one patient commented that the treatment did not seem to deal with his concerns, however this person did not complete the intervention.

To date, two other qualitative studies in different contexts have evaluated patients experiences of MCT when delivered by psychologists. All the patients diagnosed with major depressive disorder who received MCT in one study,^31^ and patients diagnosed with cancer and anxiety and depression in the other study^32^ were reported to understand MCT in a way that was consistent with the metacognitive model. Patients in these studies described developing new relationships with their thoughts, for example being in control of whether they engaged with thoughts and talked about having more effective ways of coping with problems in the future. The results of the present study indicate that group MCT is also suitable for cardiac patients, as the diverse worries of CR patients can be addressed without the requirement that the content of concerns are discussed, and techniques such as SPACE and worry postponement have ‘face validity’ as skills that can be practised. CR patients reported being able to use various group MCT techniques and perceived changes and improvements in their thinking, emotions and well-being as a result. The results support continued use of group MCT in CR and point to areas that can be monitored to avoid drift in patient understanding and implementation of techniques.

This study has raised several key considerations for the implementation of group MCT within CR and other physical therapy services. Patients noted the benefit of having CR staff deliver group MCT, despite being non-specialists in mental health. Group MCT has been found to be an effective treatment for depression and anxiety in cardiac patients,^33^ and we found some patients mis-interpreted some of the techniques (ie, SpACE). Interestingly, the SpACE technique and reviewing homework practice of SpACE was found to be an aspect of the protocol that therapists were the least adherent to in the trial.^23^ This is an important consideration for training whereby practising the implementation of the SpACE technique is required. Additionally, the group format was found to be largely positive for patients, despite a minority finding that it allowed too much time to be spent discussing other patients concerns. This also raises an important training consideration, in that CR staff may benefit from additional emphasis on managing group dynamics and maintaining the focus on discussing mental regulation rather than thought content.

This qualitative study is the first to explore CR patients’ understanding and experiences of group MCT. In the context of increasing calls for the integration of mental and physical healthcare such studies can help to investigate how complex psychological interventions such as group MCT, as delivered by non-specialists such as CR staff, are experienced and understood by patients.^34–36^ However, only four patients who did not complete group MCT were interviewed, therefore, we have limited information on the reasons why CR patients did not complete the intervention, which may be of particular importance in the context of CR, where nonattendance is widely reported.^37^

Qualitative research is unavoidably influenced by researchers own experiences and perspectives.^38 39^ In this study, the authors have drawn on their knowledge of MCT to interpret patient accounts, and address questions concerning the nature of the relationship between intervention and patient. In doing so subjectivity should be understood as a strength rather than a weakness.^40^

## Conclusions

The results of the present study support the implementation of group MCT for anxiety and depression in CR patients. Patients identified advantages to the group format linked to non-specific supportive factors and attributed specific positive value to techniques that facilitated the acquisition of new psychological perspectives on worry. Specific techniques of group MCT were viewed positively and in the main the patients view was consistent with the model, supporting the transferability of treatment to a physical health context and delivery by non-specialists. The majority of patients expressed an understanding of MCT that was consistent with the theoretical underpinnings of the model, despite the abstract and subtle nature of some of the central constructs. The results support transferability and continued evaluation of group MCT therapy in physical health settings.

## Data Availability

Qualitative data are not freely available due to the potential for patient identification upon reading of part or whole interviews transcripts. Data may be anonymised and made available on reasonable request.
