# Supplementary material for: Cardiac rehabilitation patients experiences and understanding of group metacognitive therapy: a qualitative study
Source: Open Heart. 2021 Jul 14;8(2):e001708. doi: 10.1136/openhrt-2021-001708 (PMC8281095; doi:10.1136/openhrt-2021-001708)
Supplement: Supplementary data [file openhrt-2021-001708supp001.pdf]

Supplementary file: Sample Characteristics

**Qualitative study participant number**	**Sex**	**Age Range**	**Ethnicity**	**Highest Level of Educational Qualification**	**Employment Status**	**Relationship Status**	**Cardiac Condition**	**Comorbid Health Conditions**	**History of Treatment for Anxiety and/or Depression**	**Group-MCT Sessions Attended Before Interview (CR Service)**
P01	Female	65-74	White British	Vocational qualification	Retired	Divorced	Adult congenital heart disease	Hypertension; diabetes mellitus type 2; MS; COPD; arthritis; chronic fatigue, fibromyalgia, high cholesterol	Past medication for anxiety; current medication for depression; has received counselling previously	6
P02	Male	45-54	White British	None	Unable to work due to long-term disability or ill health	Divorced	ACS; stable heart failure	Hypertension; high cholesterol; COPD	No history of relevant medication or psychological therapy	1
P03	Male	55-64	White British	GCSE or equivalent	Unable to work due to long-term disability or ill health	Divorced	Adult congenital heart disease	Hypertension	No history of relevant medication or psychological therapy	6
P04	Female	55-64	White British	Postgraduate degree	Retired	Single	ACS (MI)	COPD; IBS	Current medication for depression and anxiety; has received person-centred	6

									therapy and mindfulness therapy previously	
P05	Male	45-54	Australian	Postgraduate degree	In full time (paid) employment	Separated	ACS (MI)	Hypertension; epilepsy	No history of relevant medication or psychological therapy	6
P06	Male	55-64	White British	None	Retired	Married	ACS (MI)	Hypertension; high cholesterol; IBS	Current medication for depression and anxiety; has received counselling previously	2
P07	Male	55-64	White British	Degree	In full-time (paid) employment	Married	Coronary heart disease	None	No history of relevant medication or psychological therapy	6
P08	Male	35-44	Duel White British and New Zealand	Postgraduate degree	In full-time (paid) employment	Cohabiting	Adult congenital heart disease	Hypertension	No history of relevant medication or psychological therapy	6
P09	Male	35-44	White British	GCSE or equivalent	Unable to work due to long-term disability or ill health	Divorced	ACS (MI)	Hypertension; high cholesterol; back pain	Currently medication for depression; has received psychotherapy previously	6

P10	Female	35-44	White British	GCSE or equivalent	Temporary sick leave	Cohabiting	ACS (MI)	Arthritis	No history of relevant medication or psychological therapy	6
P11	Male	45-54	White British	Vocational qualification	Unable to work due to long-term disability or ill health	Widowed	Adult congenital heart disease	COPD; arthritis	Past medication for depression and anxiety; has never received psychological therapy	4
P12	Male	≥75	White British	Vocational qualification	Retired	Married	Atrial fibrillation	Hypertension; COPD	Current medication for depression and anxiety; has received counselling previously	2
P13	Male	55-64	White British	None	Retired	Separated	Stable heart failure	IBS or abdominal problems; arthritis	Past medication for depression; has been treated by a mental health worker previously	6
P14	Male	55-64	White British	None	In full time (paid) employment	Married	ACS (MI)	Atherosclerosis	Past medication for depression; has been treated by a	6

									psychiatrist previously	
P15	Male	55-64	White British	A level	In full-time (paid) employment	Married	ACS (MI)	COPD	No history of relevant medication or psychological therapy	4
P16	Female	≥75	White British	Diploma	Retired	Married	Stable heart failure	Hypertension; high cholesterol; arthritis; diverticulitis	Current medication for depression and anxiety; has received CBT and been treated by a psychiatrist previously	4
P17	Female	65-74	White British	Vocational qualification	Retired	Divorced	ACS (MI)	Diabetes mellitus type 2	Current medication for depression; has received counselling previously	6
P18	Male	45-54	White British	GCSE or equivalent	Unable to work due to long-term disability or ill health	Single	ACS (MI)	IBS; Fabry disease; Crohn disease; renal failure	Past medication for depression and anxiety; has been treated by a psychologist previously	5
P19	Male	55-64	White British	None	Unemployed	Single	ACS (MI)	None	No history of relevant medication or	4

									psychological therapy	
P20	Female	55-64	White British	Diploma	In part-time (paid) employment	Divorced	ACS (MI)	Hypertension; diabetes mellitus type 2; arthritis; breast cancer	Current medication for anxiety; has received CBT and counselling previously	4
P21	Male	45-54	White British	Vocational qualification	Unable to work due to long-term disability or ill health	Separated	ACS (MI)	High cholesterol	No history of relevant medication or psychological therapy	6
P22	Male	55-64	White British	Vocational qualification	In full-time (paid) employment	Married	ACS (MI)	Breathing problems or COPD; IBS; arthritis	Past medication for depression; has received victim support counselling	6
P23	Female	55-64	White British	None	Unemployed	Married	ACS (MI)	Breathing problems or COPD	No history of relevant medication or psychological therapy	6
P24	Male	45-54	White British	Vocational qualification	In full-time (paid) employment	Cohabiting	ACS (MI)	None	Past medication for depression and anxiety; has been treated by a	2

BMJ Publishing Group Limited (BMJ) disclaims all liability and responsibility arising from any reliance placed on this supplemental material which has been supplied by the author(s)

									psychologist previously	
